# Association between lean mass and adolescent idiopathic scoliosis: a meta-analysis

**DOI:** 10.1186/s12891-023-06622-4

**Published:** 2023-08-24

**Authors:** Wei Xu, Jiajie Zang, Fan Wu

**Affiliations:** 1 School of Public Health, Shanghai Jiao Tong University, Shanghai, 200025 China; 2 Shanghai Municipal Center for Disease Control and Prevention, Shanghai, 200336 China

**Keywords:** Adolescent idiopathic scoliosis, Lean mass, Etiology

## Abstract

**Objective:**

The objective of this meta-analysis was to evaluate the association between lean mass and adolescent idiopathic scoliosis (AIS).

**Methods:**

English databases CENTRAL (The Cochrane Library and the Cochrane Back Review Group Trials Register), MEDLINE, EMBASE, PubMed, Web of Science and Chinese databases CBM, CNKI, VIP, WANGFANG DATA were searched for the relevant case control studies and cross-sectional studies. Two authors selected studies and extracted data independently. Data analysis was performed by Stata15.0.

**Results:**

Eight studies were included, with a total of 1771 cases of AIS and 6340 controls. AIS group had a lower lean mass compared to control group [MD =  − 1.95, 95% CI (− 2.96, − 0.93)]. In the subgroup analysis, female AIS patients had a lower lean mass than the control group [MD =  − 1.76, 95% CI (− 2.63, − 0.88)]. The mean difference of lean mass between AIS patients and control group in studies with adults [MD =  − 3.96, 95% CI (− 7.26, − 0.67)] is much greater than studies without adults [MD =  − 1.04, 95% CI (− 1.59, − 0.49)]. There was not statistically significant in European studies [MD =  − 2.10, 95% CI (− 4.35, 0.14)], but in Asian studies lean mass in AIS patients was lower than the control group [MD =  − 2.26, 95% CI (− 3.98, − 0.54)]. Study type, gender, age, and geography condition were thought to have no effect on the primary outcome of lean mass by subgroup analysis.

**Conclusion:**

In the meta-analysis, AIS group had a lower lean mass compared to control group, which indicated that lean mass may be involved in the pathogenesis of AIS. But limited by the number of studies we included; the above conclusions need to be validated by more high-quality studies.

## Introduction

Scoliosis is defined as a three-dimensional (3D) structural deformity of the spine [1] and adolescent idiopathic scoliosis(AIS) is the most common type of scoliosis, of which the prevalence are most found in the literature is 2%-3% [2]. AIS is more prevalent in girls than in boys [1], the prevalence of AIS is also related to geography, the prevalence range was found in China was 0.62%-8.89% [3, 4]. The quality of life in AIS patients would be lower than normal people for the effects of disease [5], commonly including back pain, the progression of curve severity, pulmonary limitations and psychological disturbances.

The etiology of AIS has not been fully elucidated [2], previous studies have indicated that the causes often involve genetic, spine biomechanics, neurology, hormones, paraxial muscles and so on [1, 6, 7]. As AIS is always companied by low bone mineral density (BMD), abnormal bone quality [8] and atrophic, asymmetric paraspinal muscle [9]. Bones and muscles may be intrinsic drivers of AIS occurrence and progression [10].

Lean mass is the weight of other components of our body without fat, mainly composed of skeletal muscle, and it plays a critical role in bone growth in children and adolescents [11–13]. An increase in lean mass is associated with an increase in bone parameters [14]. Besides, lean mass is associated with skeletal muscle strength [15]. Less lean mass means less muscle mass and muscle strength. Therefore, lean mass may affect the progression of AIS by affecting the growth of bone and muscle in patients.

Recently, there were numerous studies reports on the relationship between AIS and body mass [16]. However, few studies have reported the association between lean mass and AIS, and the conclusions of the available studies are still inconsistent. The aim of this review is to find out whether there was a reduction or increase in lean mass in AIS patients compared with controls by meta-analysis, which may further clarify the relationship between lean mass and AIS.

## Methods

This review was conducted in accordance with the Preferred Reporting Items for Systematic Reviews and Meta Analyses (PRISMA) statement [17]. The protocol for this review was registered at PROSPERO (CRD42023423684).

### Data sources and searches strategy

Searches were conducted in the following databases CENTRAL (The Cochrane Library and the Cochrane Back Review Group Trials Register), EMBASE, PubMed, Web of Science, CBM, CNKI, VIP, WANGFANG DATA from database inception to 30th April 2023. All searches were peer reviewed (checked for accuracy by 2 researchers). The following search strategy were used: (1) PubMed: ((scoliosis[MeSH Terms]) or (scoliosis) or (Adolescent idiopathic scoliosis)) and ((lean mass[MeSH Terms]) or (lean mass) or (fat free mass) or (body composition)); (2) Embase: ((‘lean mass’/exp OR ‘lean mass’) or (‘fat free mass’/exp OR ‘fat free mass’) or (‘body composition’/exp OR ‘body composition’)) and ((‘scoliosis’/exp OR ‘scoliosis’) or (‘adolescent idiopathic scoliosis’/exp OR ‘adolescent idiopathic scoliosis’)); (3) Cochrane Library: ((“scoliosis”):ti,ab,kw or (“idiopathic scoliosis”):ti,ab,kw) and ((“body composition”) or (“lean body mass”) or (“fat free body mass”)):ti,ab,kw (Word variations have been searched); (4) Web of Science: ((TS = (lean mass) or TS = (fat free mass) OR TS = (body composition)) and (TS = (scoliosis) OR TS = (adolescent idiopathic scoliosis)).

### Study selection and criteria

Two reviewers independently screened studies against the inclusion criteria, assessing titles and abstracts to identify potentially relevant studies, then reviewing full texts, with disagreements resolved by discussion or by referral to a third reviewer. We included primary studies (RCTs, cohort studies, case–control studies) in English or Chinese on (1) study population: adolescent patients who were diagnosed with AIS in medical institutions by measuring Cobb angle; (2) lean mass of AIS patients and controls were measured; (3) controls were age- and gender- matched and healthy. We excluded studies which were (1) Reviews, systematic reviews, commentaries, case studies and case series (3) non-comparative studies (3) republished articles.

### Data extraction and quality assessment

Data were extracted by two authors independently from all eligible studies. The extracted information including type of study, target group, country where the study was conducted, sociodemographic and clinical characteristics, and data related to the risk-of-bias assessment.

The study quality was assessed by the Newcastle–Ottawa Scale (http://www.ohri.ca/programs/clinical_epidemiology/oxford.htm), the studies were classified into four quality levels: very low risk of biases (9 points), low risk of biases (7 to 8 points), moderate risk of biases (5–6 points), and high risk of biases (< 5 points).

### Data analysis

This meta-analysis was performed with Stata15.0. Heterogeneity among studies was assessed using the I^2^ statistic test, with *P* < 0.05 and I^2^ > 50% being assumed to indicate statistical significance. The meta-analysis was applied using a fixed-effect model if there was no statistical heterogeneity (*P* > 0.05, I2 ≤ 50%); otherwise, a random-effect model was used (*P* < 0.05, I^2^ > 50%). We conducted subgroup analyses according to tudy type, sex, age and region to explore the causes of heterogeneity. And the sensitivity analysis was performed permutation test [18] to identify whether one specific study significantly affected the data.

## Results

### Search results and characteristics of included studies

**Fig. 1:**
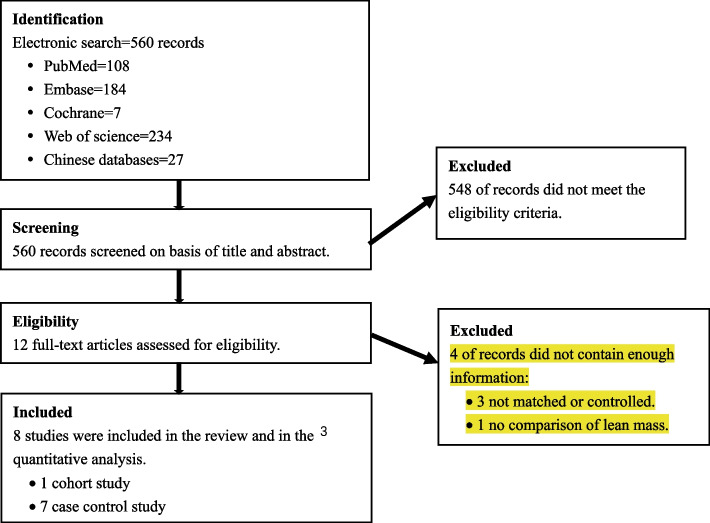
Flow chart of study selection process

We assessed a total of 560 titles and abstracts for eligibility and 548 were ineligible (Fig. 1). Twelve studies were full-text articles assessed, eight studies were included in the systematic review and mete-analysis. The characteristics of included studies and quality assessment are described in Table 1. Eight studies were identified, including seven case–control studies [19–25] and one cohort sutdy [26], which comprised a total of 1771 AIS cases and 6340 control cases in our study. All eight studies scored > 6 stars, which was considered to be high quality.

**Table 1:** Characteristics of the included studies

Study	Country	Study type	Group	N (m/f)	Age(year) MEAN(SD)	Cobb angle(°) MEAN(SD)	NOS scores
Barrios2011 [19]	Spain	Case–control	AIS	0/52	13.9(1.9)	27(20–58)	7
			Control	0/92	13.8(0.8)		
Ramírez2013 [20]	Spain	Case–control	AIS	0/27	17.4	dorsal66(15)/lumbar35(16)	8
			Control	0/488	/		
Wang2016 [21]	China	Case–control	AIS	47/0	15.3(2.2)	52.7(8.0)	8
			Control	40/0	15.3(1.8)		
Tam2016 [22]	China	Case–control	AIS	0/148	12.91(0.63)	23.5(8.9)	9
			Control	0/116	12.96(0.45)		
Zheng2017 [23]	China	Case–control	AIS	496/706	14.03(1.38)	18.68 (8.16)	9
			Control	496/706	14.03(1.38)		
Normand2022 [24]	Canada	Case–control	AIS	0/19	14.8(1.7)	27.1(10.5)	8
			Control	0/19	14.8(2.1)		
Lau2023 [25]	China	Case–control	AIS	0/10	13.71(2.04)	24.50(6.38)	7
			Control	0/12	12.11(1.92)		
Clark2014 [26]	UK	Cohort study	AIS	266	10/15^a^	/	8
			Control	4371	10/15^a^		
Total	/	/	AIS	1771	/	/	/
	/	/	Control	6340	/	/	/

*N* Number of participants

^a^Outcome was measured at 10 years old, AIS happened at 15 years old

### Meta analysis

#### Association of lean mass and AIS

**Fig. 2:**
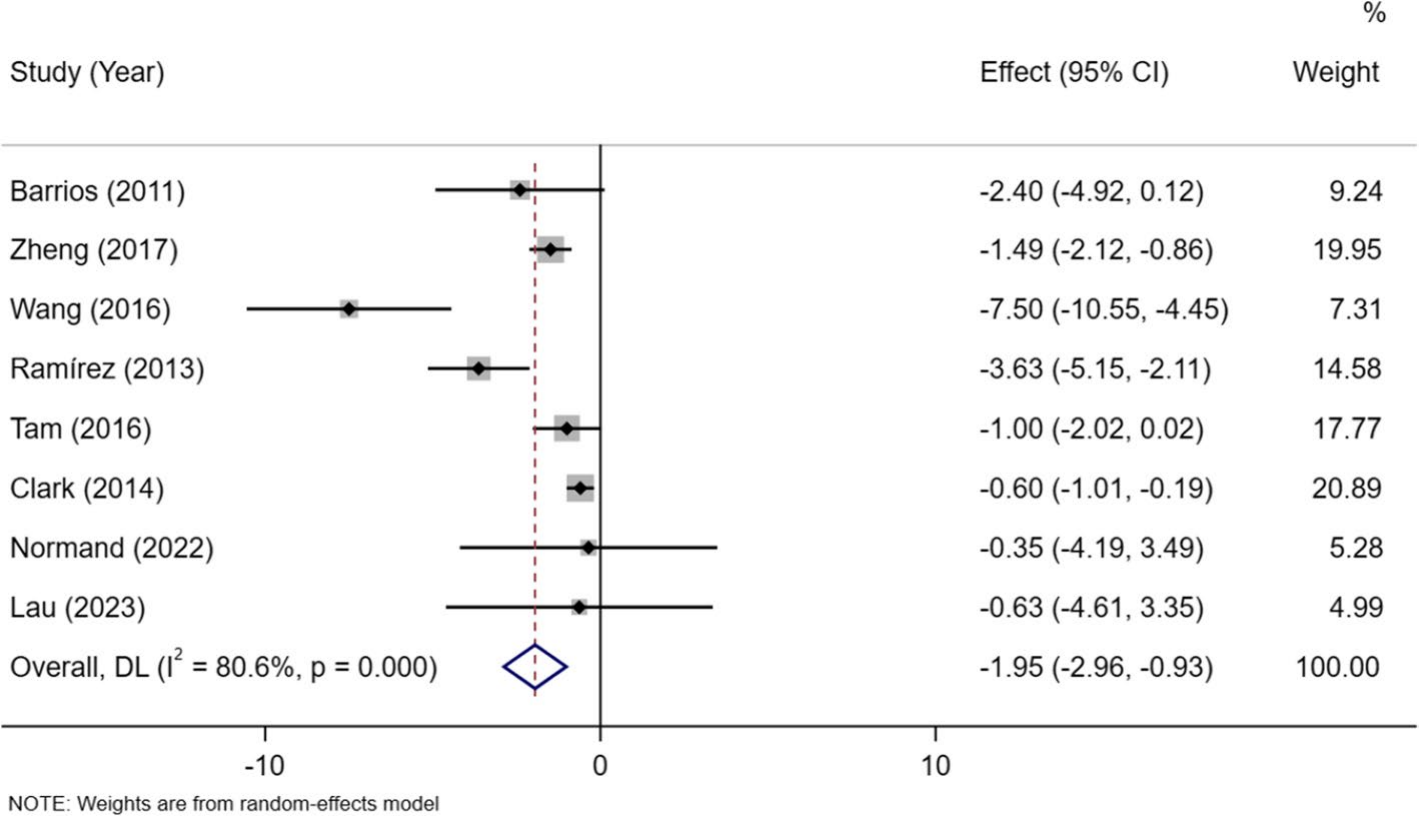
Meta-analysis of lean mass in AIS versus controls. The differences are mean differences(kg)

In total, 1771 cases and 6340 controls of eight sutdies were included in the analysis. A random-effect model was adopted there was statistical heterogeneity among the eight studies (*P* < 0.001, I^2^ = 80.6%). The results showed lower lean mass in the AIS group than the control group[MD =  − 1.95, 95% CI (− 2.96, − 0.93)] (Fig. 2).

#### Subgroup analysis

##### Study type

**Fig. 3:**
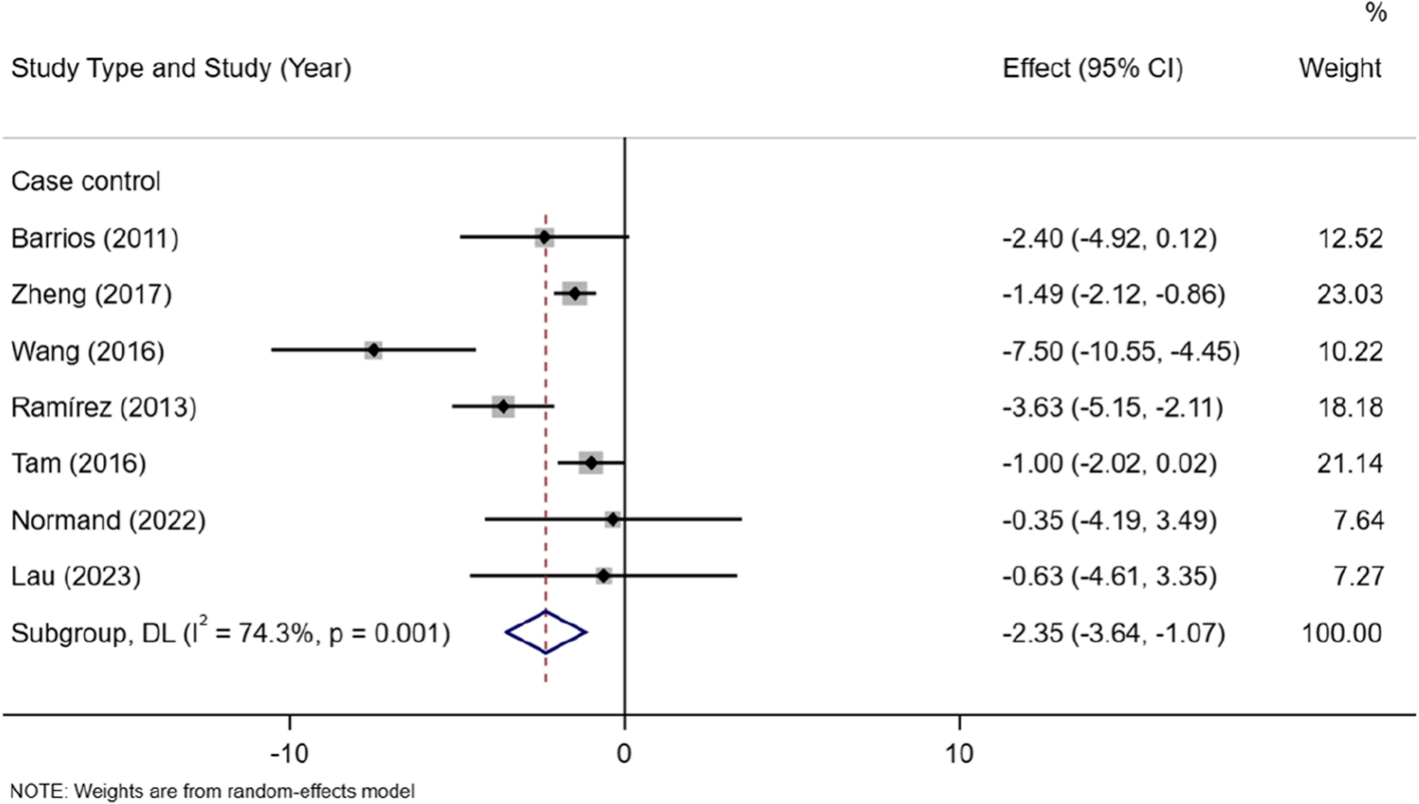
Subgroup meta-analysis of lean mass in AIS versus controls in case–control studies

There are 7 case–control studies and 1 cohort study, the analysis showed that in case–control studies lean mass in the AIS group was lower than the control group[MD =  − 2.35, 95% CI (− 3.64, − 1.07)] (I^2^ = 74.3%, *P* < 0.05) (Fig. 3).

##### Gender

**Fig. 4:**
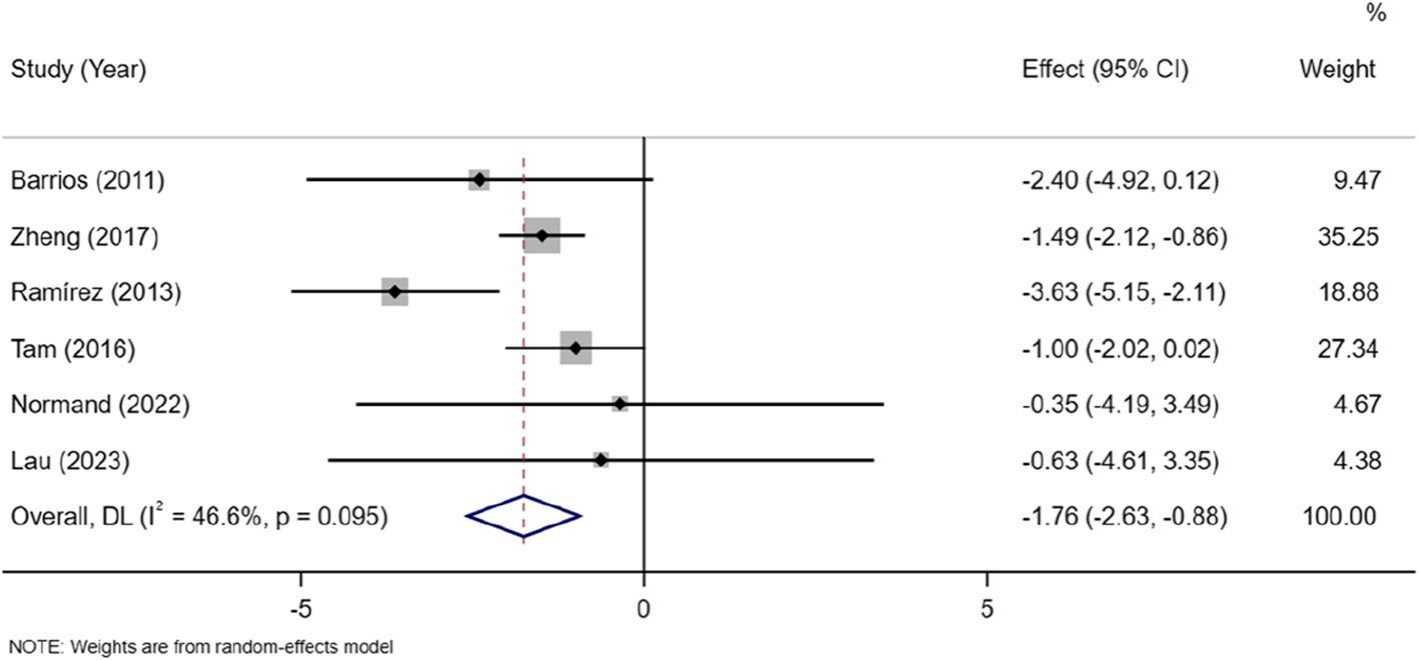
Subgroup meta-analysis of lean mass in AIS versus controls in female patients

AIS is more common in young female patients. There were 6 studies conducted in female specially. The analysis showed lower lean mass in the AIS group than the control group in female[MD =  − 1.76, 95% CI (− 2.63, − 0.88)](I^2^ = 46.6%, *P* = 0.095) (Fig. 4).

##### Age

**Fig. 5:**
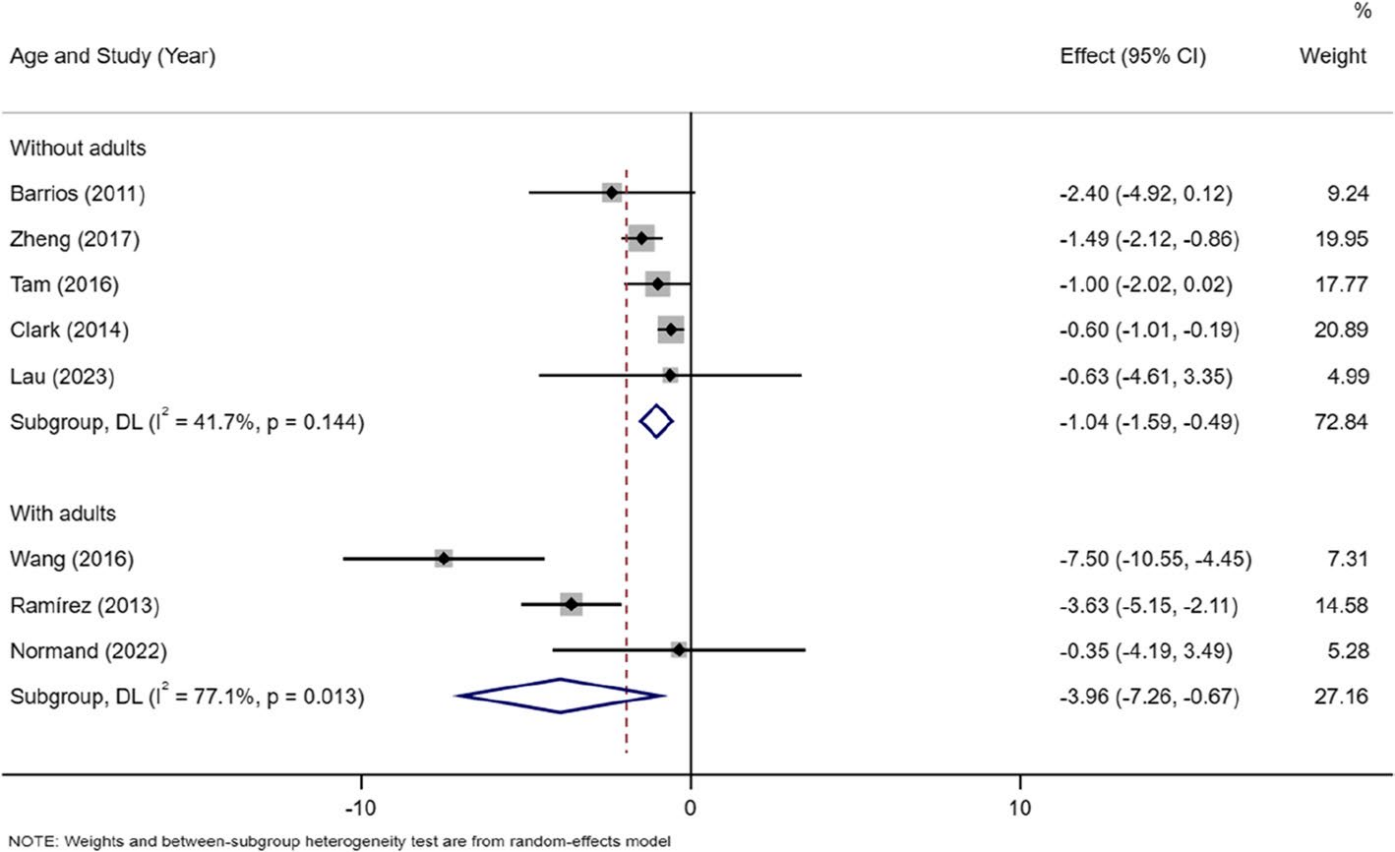
Subgroup meta-analysis of lean mass in AIS versus controls in studies included adolescents only or included both adolescents and adults

Three of the included studies included adults as sutdy subject, and others included adolescents only. The analysis showed lower lean mass in the AIS group than the control group in studies which were only with adolescents [MD =  − 1.04, 95% CI (− 1.59, − 0.49)](I^2^ = 41.7%, *P* = 0.144), also in studies with both adolescents and adults[MD =  − 3.96, 95% CI (− 7.26, − 0.67)](I^2^ = 77.1%, *P* < 0.05). Besides, there was no heterogeneity in studies without adults (Fig. 5).

##### Region

**Fig. 6:**
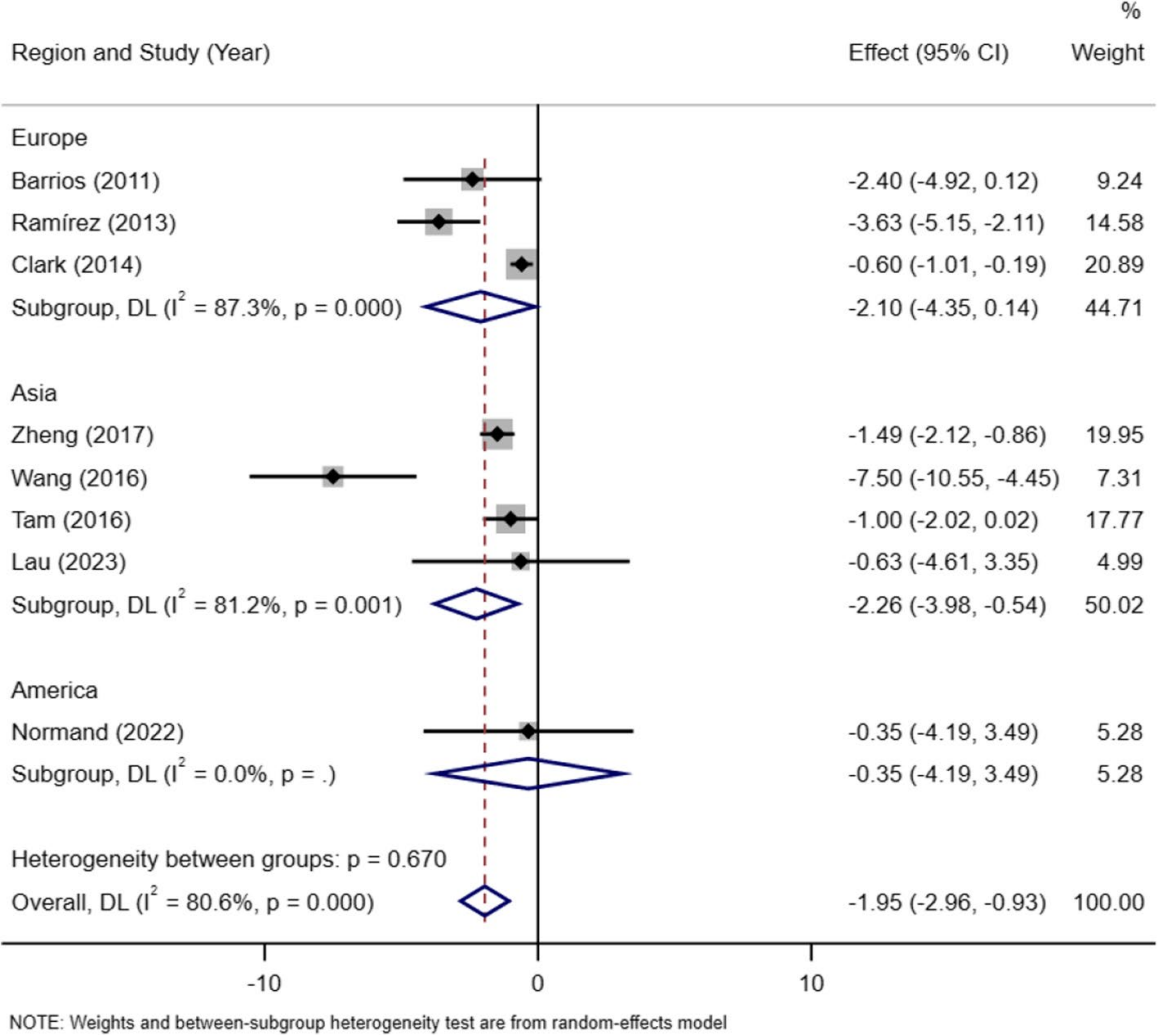
Subgroup meta-analysis of lean mass in AIS versus controls in studies in Asian countries or European countries

The subjects of 8 included studies consisted of European in 3 studies and Asian in 4 studies and America in 1 study. The subgroup analysis showed that the mean difference of lean mass between AIS patients and control group was not statistically significant in European studies[MD =  − 2.10, 95% CI (− 4.35, 0.14)]( I^2^ = 87.3%, *P* < 0.05), but in Asian studies the lean mass in AIS patients was lower than the control group[MD =  − 2.26, 95% CI (− 3.98, − 0.54)](I^2^ = 81.2%, *P* < 0.05) (Fig. 6).

#### Sensitivity analysis and publication bias

**Fig. 7:**
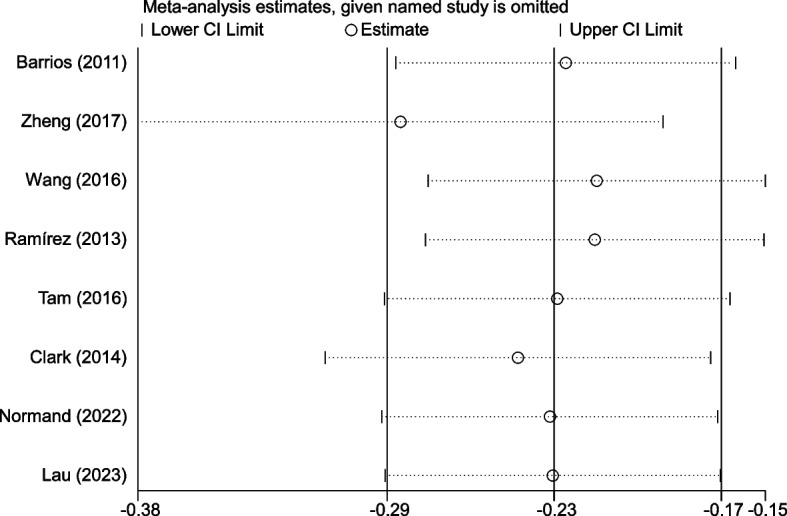
Sensitivity analysis of included studies

The results of sensitivity analysis was presented in Fig. 7. It was demonstrated that the exclusion of any single trial did not markedly effect the overall measures of the relationship between lean mass and AIS. Publication bias was not performed for there were only 8 studies incuded.

## Discussion

In this study, we analyzed the potential association between lean mass and AIS. There is a significant decrease in lean mass in AIS patients compared with healthy participants. This finding unifies the discordant findings from the 8 included studies.

In previous studies, one theory about the association between body composition and AIS is that dysfunctional paraspinal muscles can cause incongruity in spinal posture and activity control, which would lead to the development of AIS through biomechanics-related mechanisms [27, 28]. In a meta analysis, small-sized associations were found between variables of balance and lower-extremity muscle strength/power, and significantly larger but still small-sized associations were found between measures of dynamic steady-state balance and maximal strength in children compared with young and old adults [29]. Lean mass is mainly composed of skeletal muscle, and greater lean mass was found correlated with significantly greater postural control in relation to balance variables in young adults [30]. A standing balance test study in AIS girls showed that the sway area was always larger in the scoliosis group than the able-bodied group. And the AIS ectomorphic girls in the study had a tendency to lean further back than a comparable able-bodied group, which could increase the risk of spinal deformity progression [31]. Therefore, less lean mass may cause posture unbalanced and finally increase the risk of progression of AIS.

Lean mass also involves in the etiology and progression of AIS according to affect the growth of bone. Lean mass plays an important role in the growth of bone in adolescents, it has a positive impact on bone density [32]. Increasing lean mass in children may help optimize bone acquisition [11]. Previous studies have shown that AIS was associated with osteopenia [33, 34]. In Chengs’ study, the result indicated that the presence of osteopenia before the presence of spinal deformity and supports the possible hypothesis that osteopenia in AIS patients might be one of the primary triggers of the spinal deformity [35]. In addition, previous studies also found lean mass is related with the curve severity in AIS patiens. Lean mass index [36] and the percentage of lean mass [37] in AIS severe group were less than mild or moderate group, which indicated that decreasing lean mass is also related to the development of AIS.

Heterogeneity was found to exist with the meta analysis. Included studies adopted different research objects, research designs, and all of which may contribute to the significant between-study heterogeneity. Therefore, we conducted a subgroup meta-analysis according to these facors. In case control studies, the mean difference of lean mass between AIS patients and control group s was greater than the meta analysis. The cohort study measured lean mass of subjects when they were 10 years old, which resulted in the mean kg and the mean difference of lean mass in this study were less than other studies. Considering the prevalence of AIS is higher in female than male and men’s lean mass is higher than women’s in both AIS patients and healthy people [38, 39], we assumed sex as one source of the heterogeneity. And the result of female subgroup meta analysis showed the mean difference of lean mass between AIS patients and control group in this subgroup analysis was greater than the meta analysis, and the heterogeneity decreased in this subgroup. Moreover, in consideration of the differences in body size between adults and adolescents, we performed a subgroup analysis of studies including adults and studies without adults. There is not significantly heterogeneity observed among studies without adluts, and the mean difference of lean mass between AIS patients and control group in studies with adults is much greater than studies without adults. This finding indicates that the change of lean mass associated with the progression of AIS are much smaller in adolescents than in adults. In the region subgroup analysis, we found there is no difference of lean mass between AIS patients and control group in European studies, but in Asian studies lean mass in AIS patients was lower than the control group. After careful analysis of these documents, we found that the gender and age of research objects were potential contributors to heterogeneity.

### Limitations

There are several limitations should be acknowledged. First, seven of the included studies were case–control study, the evidence may be more reliable if there were RCTs included in our study. Second, the heterogeneity is significant, which arises owing to differences across studies in sample size (small sample), design or conduct, geographic region, and population characteristics among other factors(sex and age). Based on the results of the heterogeneity test a random-effect model was adopted in the meta-analysis. However, choosing a model based on the result of the heterogeneity test is inappropriate, because the heterogeneity is consist of clinical heterogeneity, methodological heterogeneity and statistical heterogeneity, and when the statistical heterogeneity is not significant, the heterogeneity may be ignored. But the results of subgroup meta analysis and sensitivity analysis revealed that meta-analysis results were consistent and stable and not affected by the heterogeneity. Third, we have included studies only published in English and Chinese, which may introduce a language bias and lead to erroneous conclusions. But previous study provides no evidence that language restricted meta-analyses lead to biased estimates of intervention effectiveness [40, 41]. Finally, due to the incomplete research data, there is a lack of study on the dose–response relationship between decreasing lean mass and Cobb’s angle in AIS patients.

## Conclusions

In conclusion, our meta-analysis showed the lean mass in AIS patients was 1.95 kg less than healthy participants, more lean mass may be a protect factor of AIS. Whether these changes are related to abnormal growth or are subsequent to nutritional or physical activity changes in AIS still remains uncertain. It must be the subject of subsequent studies.

## Data Availability

The datasets used and/or analysed during the current study are available from the corresponding author on reasonable request.
